# Supplementary material for: Time-series transcriptome analysis identified differentially expressed genes in broiler chicken infected with mixed Eimeria species
Source: Front Genet. 2022 Aug 8;13:886781. doi: 10.3389/fgene.2022.886781 (PMC9393255; doi:10.3389/fgene.2022.886781)
Supplement: Supplementary file 2 [file DataSheet1.ZIP › 7dpi_GO.Gsea.1625106495923/GOCC_PROTON_TRANSPORTING_V_TYPE_ATPASE_COMPLEX.html]

Details for gene set GOCC\_PROTON\_TRANSPORTING\_V\_TYPE\_ATPASE\_COMPLEX[GSEA]

|  || Dataset | TMM\_7dpi\_gct\_format\_7dpi\_gct\_format.Class\_4dpi.cls #PC\_versus\_NC.Class\_4dpi.cls #PC\_versus\_NC\_repos |
| Phenotype | Class\_4dpi.cls#PC\_versus\_NC\_repos |
| Upregulated in class | 1 |
| GeneSet | GOCC\_PROTON\_TRANSPORTING\_V\_TYPE\_ATPASE\_COMPLEX |
| Enrichment Score (ES) | 0.8716581 |
| Normalized Enrichment Score (NES) | 3.0805016 |
| Nominal p-value | 0.0 |
| FDR q-value | 0.0 |
| FWER p-Value | 0.0 |
Table: GSEA Results Summary

  

Fig 1: Enrichment plot: GOCC\_PROTON\_TRANSPORTING\_V\_TYPE\_ATPASE\_COMPLEX      
 Profile of the Running ES Score & Positions of GeneSet Members on the Rank Ordered List

  

| SYMBOL | TITLE | RANK IN GENE LIST | RANK METRIC SCORE | RUNNING ES | CORE ENRICHMENT || 1 | ATP6V1C2 | na | 4 | 2.128 | 0.1110 | Yes |
| 2 | ATP6V0A4 | na | 19 | 1.686 | 0.1979 | Yes |
| 3 | ATP6AP1 | na | 21 | 1.657 | 0.2845 | Yes |
| 4 | ATP6V0D2 | na | 24 | 1.636 | 0.3699 | Yes |
| 5 | ATP6V0C | na | 30 | 1.603 | 0.4533 | Yes |
| 6 | ATP6AP2 | na | 43 | 1.481 | 0.5297 | Yes |
| 7 | ATP6V1D | na | 46 | 1.458 | 0.6058 | Yes |
| 8 | ATP6V0E1 | na | 72 | 1.354 | 0.6746 | Yes |
| 9 | ATP6V1H | na | 80 | 1.313 | 0.7426 | Yes |
| 10 | ATP6V0B | na | 100 | 1.252 | 0.8065 | Yes |
| 11 | ATP6V1G3 | na | 102 | 1.247 | 0.8717 | Yes |
| 12 | ATP6V1G1 | na | 2444 | 0.199 | 0.6868 | No |
| 13 | TCIRG1 | na | 3866 | 0.020 | 0.5693 | No |
| 14 | ATP6V1C1 | na | 4112 | -0.007 | 0.5493 | No |
| 15 | ATP6V0A1 | na | 4522 | -0.052 | 0.5179 | No |
| 16 | ATP6V0D1 | na | 5522 | -0.151 | 0.4425 | No |
| 17 | ATP6V1A | na | 5917 | -0.192 | 0.4197 | No |
| 18 | ATP6V1B2 | na | 5973 | -0.199 | 0.4255 | No |
| 19 | ATP6V0E2 | na | 6836 | -0.290 | 0.3688 | No |
| 20 | TMEM199 | na | 9274 | -0.595 | 0.1966 | No |
| 21 | ATP6V0A2 | na | 9318 | -0.602 | 0.2245 | No |
Table: GSEA details [plain text format]

  

Fig 2: GOCC\_PROTON\_TRANSPORTING\_V\_TYPE\_ATPASE\_COMPLEX      
 Blue-Pink O' Gram in the Space of the Analyzed GeneSet

  

Fig 3: GOCC\_PROTON\_TRANSPORTING\_V\_TYPE\_ATPASE\_COMPLEX: Random ES distribution      
 Gene set null distribution of ES for **GOCC\_PROTON\_TRANSPORTING\_V\_TYPE\_ATPASE\_COMPLEX**
